# Supplementary material for: Chlamydia trachomatis modulates the expression of JAK-STAT signaling components to attenuate the type II interferon response of epithelial cells
Source: mBio. 2024 Aug 28;15(10):e01834-24. doi: 10.1128/mbio.01834-24 (PMC11481910; doi:10.1128/mbio.01834-24)
Supplement: Supplemental Figures — Figures S1 to S5. [file mbio.01834-24-s0001.pdf]

Figure S1

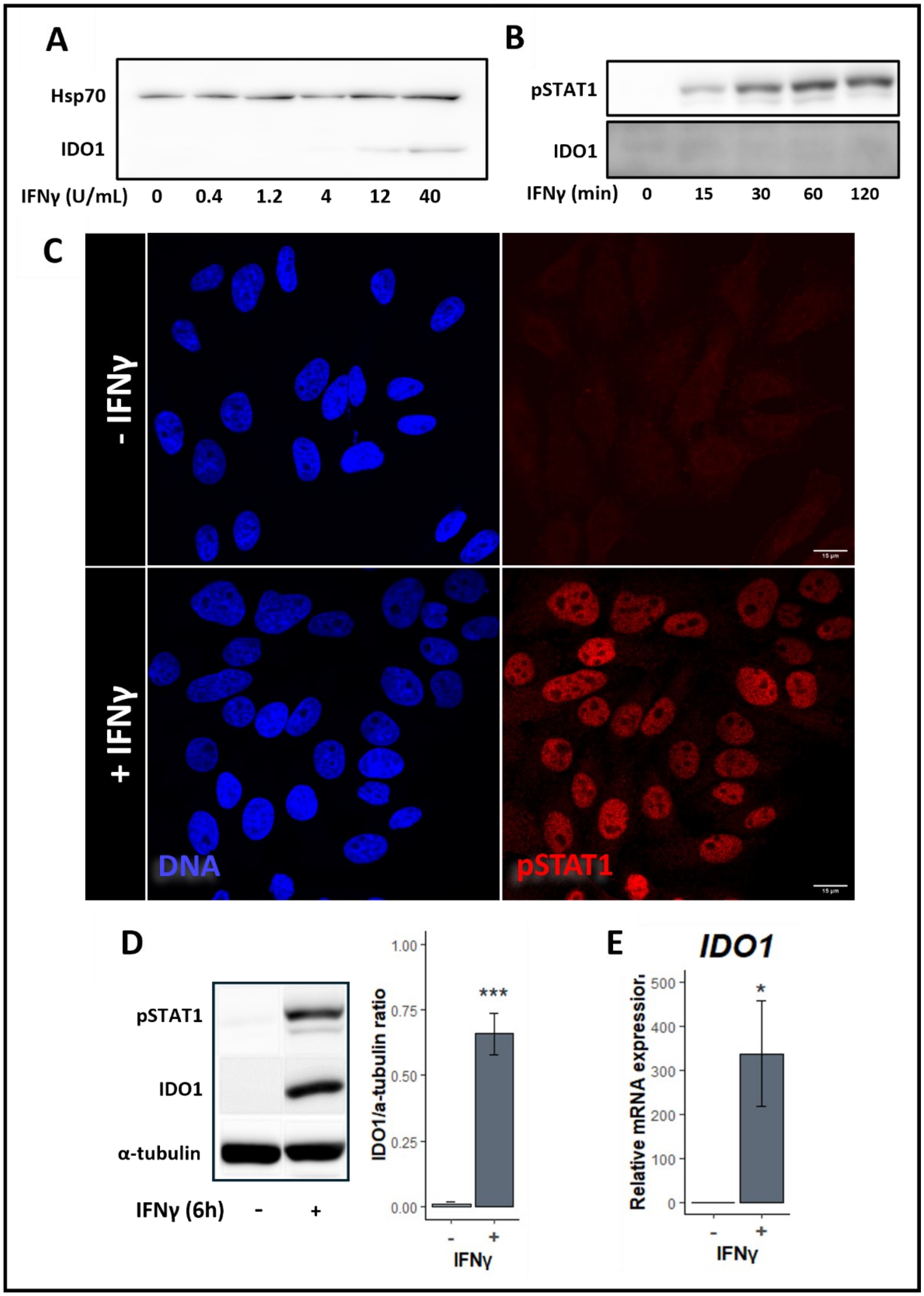
A
Hsp70
IDO1
IFNγ (U/mL) 0 0.4 1.2 4 12 40
B
pSTAT1
IDO1
IFNγ (min) 0 15 30 60 120
C
- IFNγ
+ IFNγ
DNA
pSTAT1
D
pSTAT1
IDO1
α-tubulin
IFNγ (6h) - +
IDO1/a-tubulin ratio
1.00
0.75
0.50
0.25
0.00
***
- +
IFNγ
E
IDO1
Relative mRNA expression
500
400
300
200
100
0
*
- +
IFNγ

Figure S2

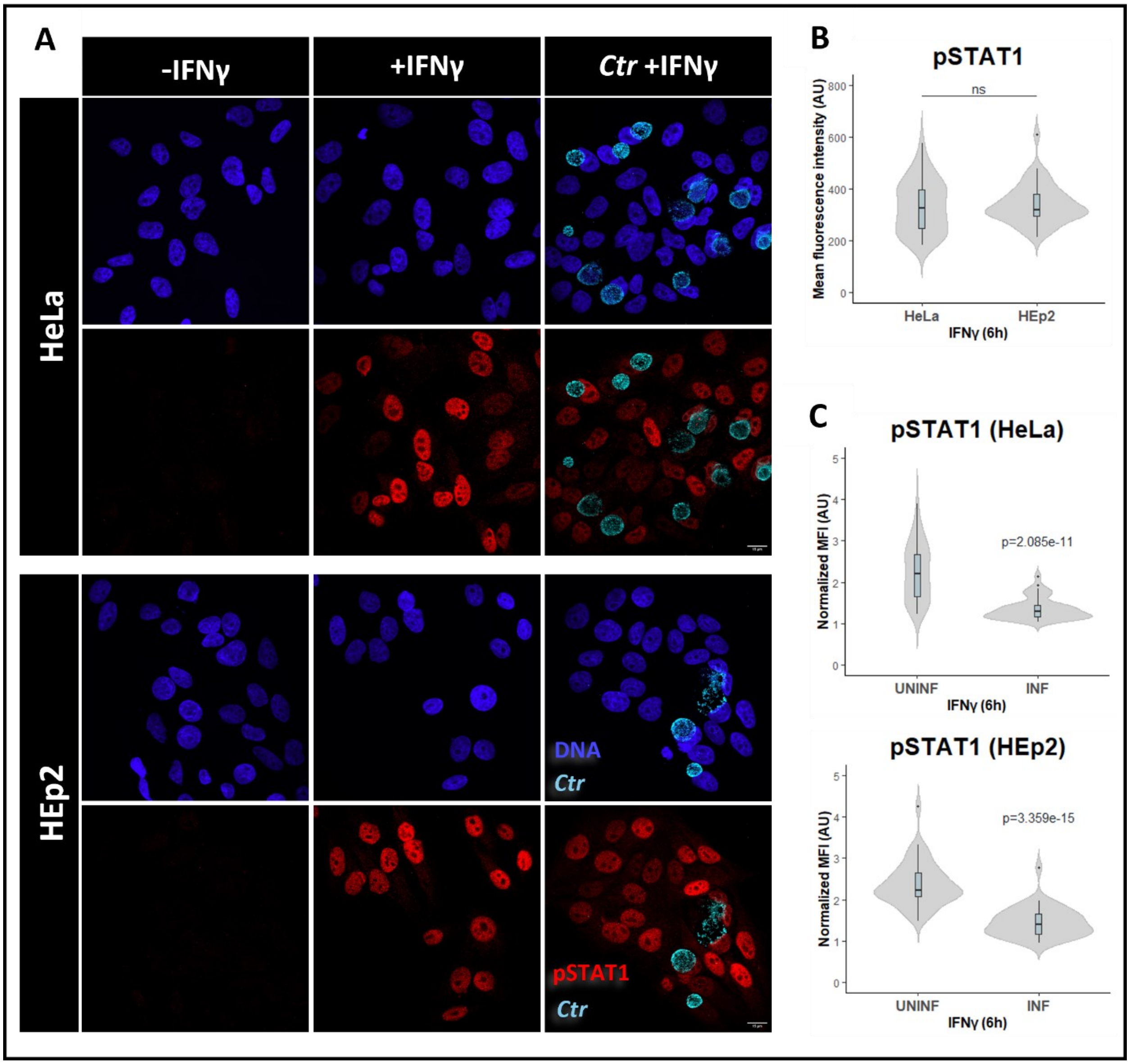
A
-IFNγ
+IFNγ
Ctr +IFNγ
HeLa
HEp2
DNA
Ctr
pSTAT1
Ctr
B
pSTAT1
Mean fluorescence intensity (AU)
800
600
400
200
0
ns
HeLa
HEp2
IFNγ (6h)
C
pSTAT1 (HeLa)
Normalized MFI (AU)
5
4
3
2
1
0
p=2.085e-11
UNINF
INF
IFNγ (6h)
pSTAT1 (HEp2)
Normalized MFI (AU)
5
4
3
2
1
0
p=3.359e-15
UNINF
INF
IFNγ (6h)

Figure S3

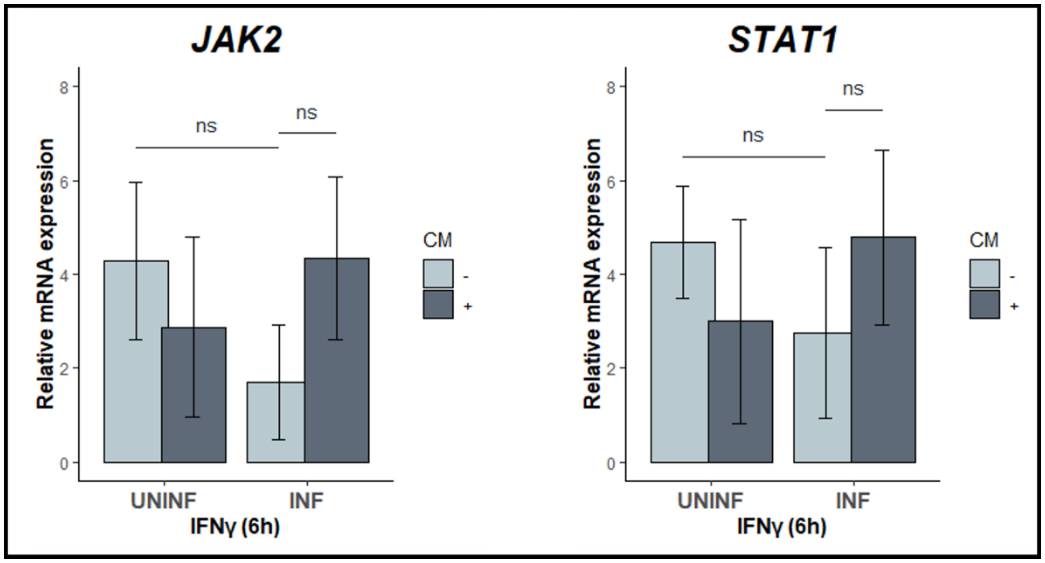
JAK2
STAT1
Relative mRNA expression
ns
ns
ns
ns
0
2
4
6
8
UNINF
INF
IFNγ (6h)
CM
-
+

Figure S4

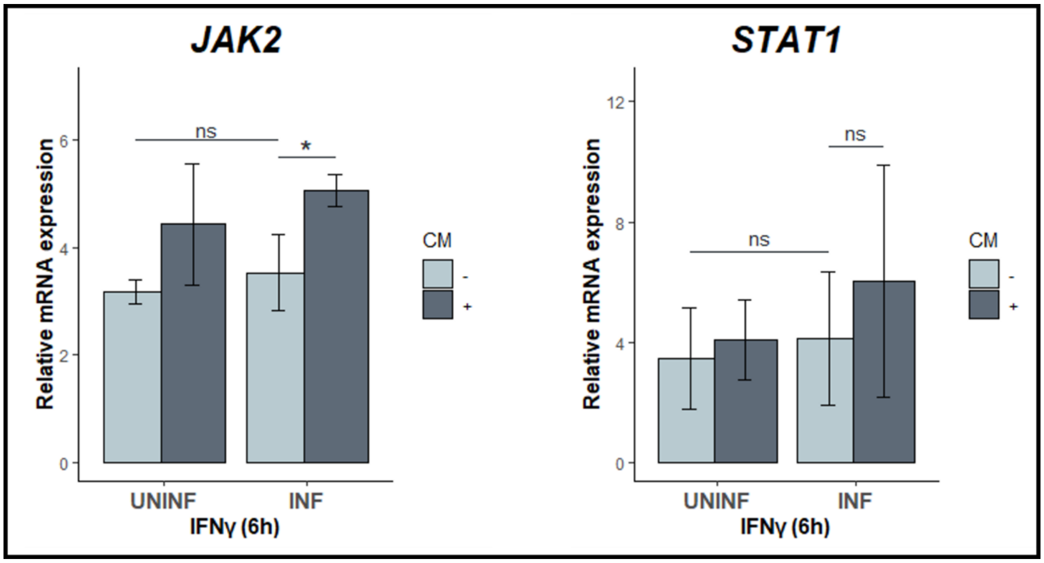
JAK2
STAT1
Relative mRNA expression
ns
*
CM
-
+
UNINF
INF
IFNγ (6h)
0
2
4
6
8
12

Figure S5

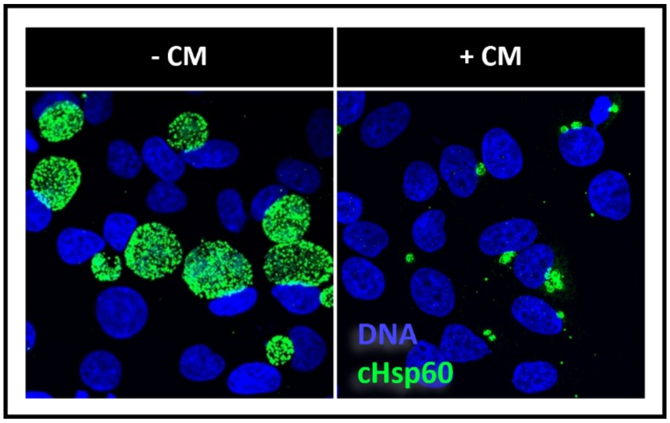

Supplemental Figure Legends

Figure S1. Optimization of IFNγ-treatment of Hep2 cells to detect activation of STAT1 and expression of the downstream target IDO1. (A) Optimizing IFN-gamma concentration to induce IDO1 expression under physiological range, HSP70 serves as a non-responsive loading control. **(B)**Immunoblot analysis of activated STAT1 (pSTAT1) and IDO1 following exposure to 10 U/mL of IFNγ at different exposure lengths. **(C)** Immunofluorescence assay showing nuclear localization of activated STAT1 in the presence of IFNγ **(D)** Immunoblot and densitometric analysis of IDO1 protein **(E)** RT-qPCR of transcript expression levels of IDO1 following treatment with IFNγ at 10 U/mL at 24h hpi for 6 hours. All data are representative of 4 independent experiments, and statistical significance was determined using Welch's t-test. $**p < 0.01$, $*p < 0.05$, ns = not significant. Scale bars at 15 μm.

Figure S2. IFNγ-induced activation of STAT1 in HeLa and HEp2 cells. (A) Representative micrographs of uninfected and infected HeLa and HEp2 cells showing nuclear localization of activated STAT1 following IFNγ treatment (**B**) Quantification of activated STAT1 raw mean fluorescence in uninfected HeLa and HEp2 cells. **(C)** Quantification of normalized activated STAT1 mean fluorescence comparing IFNγ-treated uninfected and infected cells for both cell lines. All data are representative of 3 independent experiments with a total of 45 nuclei measured per group. Statistical significance was determined using Welch's t-test. ns = not significant. Scale bars at 15 μm.

Figure S3. *C. trachomatis* serovar D downmodulates expression of JAK2 and STAT1 expression in infected HEp2 cells. RT-qPCR analysis of JAK2 and STAT1 transcript expression in HEp2 cells that infected with the genital serovar at MOI=4. Chloramphenicol (CM) was added at 8 hpi, while 20 U/mL IFNγ was added for 6h starting at 24hpi prior to RNA collection. All data are representative of 3 independent experiments, and statistical significance was determined using Welch's t-test. ns = not significant.

Figure S4. *C. trachomatis* serovar L2 downmodulates expression of JAK2 and STAT1 transcript in infected End1 cells. RT-qPCR analysis of JAK2 and STAT1 transcript expression in endocervical End1 cells that are infected with *C. trachomatis* lymphogranuloma venereum (LGV) serovar L2 at an MOI=4. Chloramphenicol (CM) was added at 8 hpi, while 20 U/mL IFNγ was added for 6h starting at 24hpi prior to RNA collection. All data are representative of 4 independent experiments, and statistical significance was determined using Welch's t-test. $**p < 0.01$, $*p < 0.05$, ns = not significant.

Figure S5. Chloramphenicol treatment limits chlamydial growth. Immunofluorescence visualization of 24hpi *chlamydial* inclusions following chloramphenicol treatment (CM) at 8 hpi observed by staining with chlamydial Hsp60 (cHSP60) antibody in HEp2 cells.
